# Renal improvement and remission in a patient with refractory ANCA-associated vasculitis treated with avacopan

**DOI:** 10.1007/s40620-023-01614-y

**Published:** 2023-04-10

**Authors:** Luis Alvarez, Neeraja Kambham, Robert Su

**Affiliations:** 1 Division of Nephrology, Department of Medicine and Nephrology, Palo Alto Medical Foundation, Palo Alto, CA USA; 2 Division of Renal Pathology, Stanford University, Stanford, CA USA; 3 Division of Rheumatology, Sutter Health System, Fremont, CA USA

**Keywords:** Avacopan, Acute renal failure, ANCA-associated vasculitis, Remission

## Abstract

Antineutrophil cytoplasmic autoantibody (ANCA)-associated vasculitis is associated with end-organ damage resulting in significant morbidity and mortality. Most recently, avacopan, an orally administered selective antagonist of the C5a receptor, was approved by the US Food and Drug Administration as an adjunctive treatment of adult patients with severe, active ANCA-associated vasculitis (granulomatosis with polyangiitis and microscopic polyangiitis) in combination with standard therapy including glucocorticoids. This case study describes a 58-year-old Asian female with severe ANCA-associated vasculitis and acute renal failure who responded to adjunctive therapy with avacopan despite being refractory to rituximab and glucocorticoid therapy.

## Introduction

Granulomatosis with polyangiitis (GPA) and microscopic polyangiitis (MPA) are the most common clinical phenotypes of antineutrophil cytoplasmic autoantibody (ANCA)-associated vasculitis that may result in end-organ damage and significant morbidity and mortality [1, 2]. Over the last two decades considerable progress has been made in the treatment of ANCA-associated vasculitis; however, the toxic nature of immunosuppressant therapies may lead to serious adverse events. Therapeutics specifically targeting the pathogenesis of the ANCA-induced inflammatory cascade continue to be developed. Most recently, avacopan, an orally administered selective antagonist of the C5a receptor (C5aR) [3, 4], was approved by the US Food and Drug Administration in October 2021 as an adjunctive treatment for adult patients with severe, active ANCA-associated vasculitis in combination with standard therapy including glucocorticoids. Avacopan has been studied in three clinical trials in patients with ANCA-associated vasculitis including two Phase 2 trials and one Phase 3 trial (ADVOCATE) [4–6]. In all three trials, patients receiving avacopan showed improved estimated glomerular filtration rate (eGFR), early rapid reduction in albuminuria, and improved health-related quality of life measures with a significant reduction in glucocorticoid use [4–6]. The ADVOCATE trial demonstrated noninferiority of avacopan to a prednisone taper in achieving remission at week 26 and superiority in maintaining remission at week 52 [4]. Additionally, patients receiving avacopan experienced a reduced relapse rate [4]. As part of an early access program, 18 patients with difficult-to-treat disease and/or rapidly progressive glomerulonephritis were treated with avacopan. Seventeen of the 18 patients achieved remission, two patients for the first time since their diagnosis [7–9]. Finally, one additional case report described a patient with ANCA-associated vasculitis who discontinued dialysis 10 days after starting avacopan [10].

This case study describes a patient with severe ANCA-associated vasculitis and acute renal failure who appears to have responded to adjunctive therapy with avacopan despite being refractory to rituximab and glucocorticoid therapy.

## Case report

**Fig. 1:**
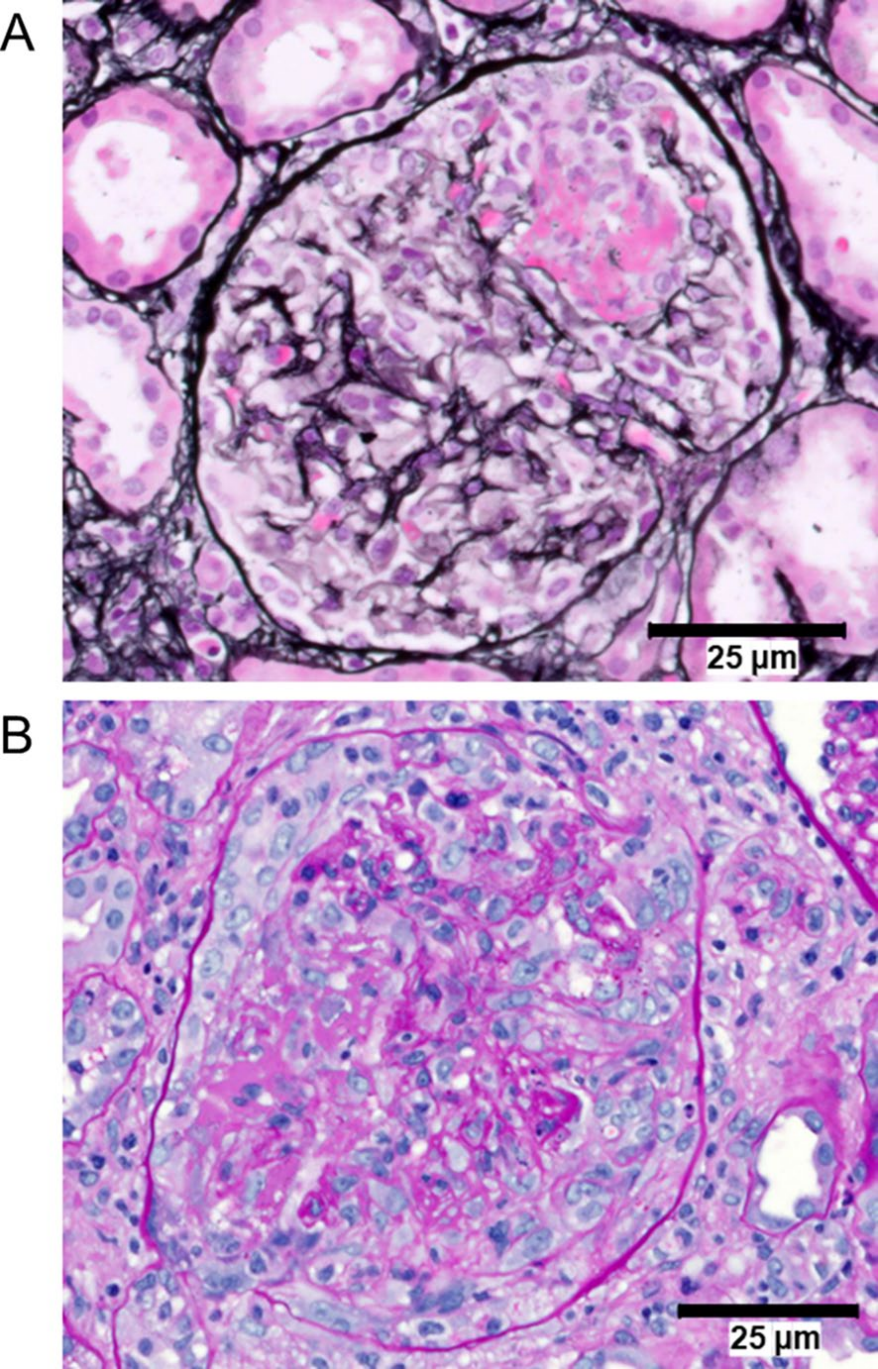
Renal biopsy. **A** Glomerulus with fibrinoid necrosis characterized by basement membrane rupture and extravasated fibrin (JMS stain, ×400); **B** glomerular necrosis with a circumferential cellular crescent (PAS, ×400)

A 58-year-old previously healthy Asian female was referred to rheumatology after experiencing joint pain (Table 1), which was unresolved with ibuprofen. Baseline eGFR of 78 mL/min/1.73 m^2^ and a serum creatinine (SCr) of 0.83 mg/dL were recorded (Day − 109 relative to avacopan initiation). The patient was initially treated with prednisone 20 mg daily for presumptive diagnosis of possible polymyalgia rheumatica (Day − 66). The patient continued to develop fatigue, headaches, ear fullness, and gastrointestinal symptoms in addition to the unresolved joint pain. Weeks later, the patient presented to the emergency room and was hospitalized (Days − 49 to − 40) with acute kidney injury (AKI) and left lower extremity deep vein thrombosis. Relevant abnormal labs and signs included: eGFR of 12 mL/min/1.73 m^2^, SCr of 3.87 mg/dL, urinary protein:creatinine ratio of 7 g/g, and microscopic hematuria. A kidney biopsy demonstrated active disease with 60% cellular crescents and glomerular necrosis lesions (Fig. 1) with minimal chronicity of 3% global glomerulosclerosis and 10% tubular atrophy and interstitial fibrosis (Day − 46); ANCA titers were detected via enzyme-linked immunosorbent assay (> 1:1280) to myeloperoxidase and a diagnosis of MPA with rapidly progressive glomerulonephritis was made.

**Table 1:** Patient timeline for the case of a 58-year-old Asian female

Days relative to avacopan initiation	Medical care setting	Symptoms	Diagnoses	Peak SCr (mg/dL) during visit	eGFR (mL/min /1.73 m^2^)	Treatments	Outcomes
− 109	Primary care	Diffuse joint pain	Joint pain	0.83	78	Ibuprofen	Joint pain unresolved
− 66	Referred to rheumatology	Joint pain	Polymyalgia Rheumatica			Prednisone 20 mg daily PO	Pain unresolved, frustrated by GC side effects (mostly GI), unable to taper
− 49 to − 40	ER visit admitted to hospital	Joint pain Fatigue Headache Ear fullness GI	AKI with fluid overload Urine analysis: microscopic hematuria with red cell casts ANCA titer results: MPO + (115 AU/mL) ANCA-associated vasculitis IFA Pattern: p-ANCA, Titer: > 1:1280 Kidney biopsy results: Rapidly progressive glomerulonephritis 60% cellular crescents and necrotizing vascular lesions, 10% IFTA, negative IgG, IgA, IgM	3.87	12	Methylprednisolone 1 g IV × 3 days Rituximab 1 g IV Methylprednisolone 50 mg daily PO Others: Furosemide diuresis Atovaquone for PCP prophylaxis Warfarin for DVT	Kidney biopsy resulting in ANCA-associated vasculitis diagnosis SCr improvements to 2.15 mg/dL Patient discharged
− 30 to − 19	Re-admitted to hospital	Rising SCr	ANCA-associated vasculitis Worsening AKI with fluid overload Partially occlusive femoral DVT New onset DM HTN Hyperkalemia Hyponatremia	3.41	14	Methylprednisolone 500 mg × 3 days Rituximab 1 g IV Methylprednisolone 50 mg daily PO Others: Furosemide diuresis Atovaquone for PCP prophylaxis Warfarin for DVT	Joint pain, fever, and chills resolved, lessened GI symptoms SCr improvements to 1.97 mg/dL Patient discharged
− 11	Renal consult for biopsy proven ANCA-associated vasculitis	Cushingoid appearance Hirsutism	ANCA-associated vasculitis Chronic residual renal insufficiency secondary to hyperparathyroidism, at risk for DVT	2.38	22	Methylprednisolone 50 mg daily PO Others: Furosemide diuresis Atovaquone for PCP prophylaxis Losartan for HTN Insulin for Type 2 DM	Patient to start avacopan and wean steroids if tolerable
*Avacopan therapy added to treatment*							
0	Nephrology clinic visit	Feels edema is improving without further use of diuretics GI symptoms Feels she has had muscle mass loss Weakness	ANCA-associated vasculitis Residual renal insufficiency Type 2 DM HTN	2.63	19	Avacopan 30 mg BID Methylprednisolone, gradual taper starting at 50 mg Others: Atovaquone for PCP prophylaxis Losartan for HTN Insulin for Type 2 DM	Started avacopan, plans for continued follow up
23	Nephrology clinic visit ~ 1 month post-avacopan	Patient demonstrating steady improvement on current regimen, still tapering steroids	ANCA-associated vasculitis Residual renal insufficiency Type 2 DM HTN	1.70	33	Avacopan 30 mg BID Methylprednisolone 40 mg Others: Atovaquone for PCP prophylaxis Losartan for HTN Insulin for Type 2 DM	Steady improvement No hospitalizations since starting avacopan
312	Nephrology clinic visit ~ 10 months post-avacopan	Further eGFR improvement	ANCA-associated vasculitis	1.15	55	Avacopan 30 mg BID	The patient has demonstrated sustained disease remission
387	Nephrology clinic visit ~ 12 months post-avacopan	uPCR undetectable	ANCA-associated vasculitis	1.02	63	Avacopan 30 mg BID	Continual improvement

*AKI* acute kidney injury, *ANCA* antineutrophil cytoplasmic autoantibody, *BID* bis in die (twice daily), *DM* diabetes mellitus, *DVT* deep vein thrombosis, *eGFR* estimated glomerular filtration rate, *ER* emergency room, *GC* glucocorticoid, *GI* gastrointestinal, *HTN* hypertension, *IFTA* interstitial fibrosis and tubular atrophy, *IV* intravenous, *MPO* myeloperoxidase, *PCP* pneumocystis pneumonia, *PO* per os (by mouth), *PRN* pro re nata (as needed), *SCr* serum creatinine, *uPCR* urinary protein:creatinine ratio

**Fig. 2:**
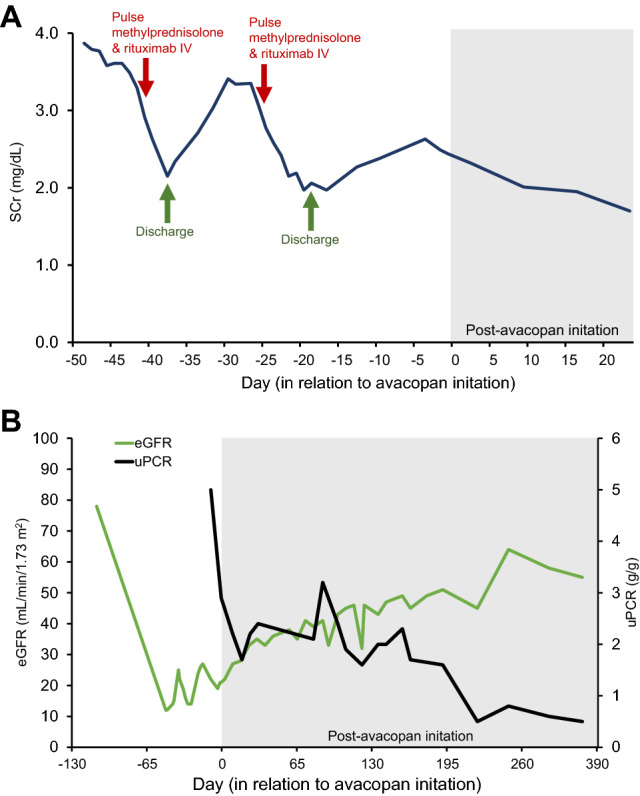
Renal function changes over time. **A** Serum creatinine levels during two hospitalizations and shortly after avacopan initiation; **B** rapid renal improvement as measured by eGFR and uPCR levels post-avacopan. *eGFR* estimated glomerular filtration rate, *IV* intravenous, *SCr* serum creatinine, *uPCR* urinary protein:creatine ratio

During the initial hospitalization (Days − 49 to − 40), treatment included pulse methylprednisolone (1 g intravenous (IV) daily for 3 days, Days − 45 to − 43), and 1 g IV rituximab (Day − 40) (Fig. 2A). SCr down trended to 2.15 mg/dL following aforementioned induction therapy, and patient was discharged on methylprednisolone 50 mg daily. Attempts to taper the methylprednisolone below 40 mg resulted in clinical deterioration (recurrent AKI and joint pain). Within two weeks from discharge, the patient developed complications due to glucocorticoid adverse events, including type 2 diabetes mellitus, hypertension, and fluid overload, requiring further treatment.

Despite initial induction therapy and outpatient methylprednisolone 50 mg daily, the patient’s SCr increased after discharge and she re-presented to the hospital with worsening AKI (SCr = 3.41 mg/dL). The patient received pulse methylprednisolone (500 mg IV daily for 3 days, Day − 30 to − 28) again and a second course of rituximab (1 g IV) (Day − 25). SCr down trended to nadir of 1.97 mg/dL during second hospitalization (Days − 30 to − 19). Upon discharge the patient resumed methylprednisolone 50 mg daily. CD20 + lymphocytes and CD19 + lymphocytes were undetectable at 0%. Although CD20 B cell depletion was confirmed, over the course of two and a half weeks SCr continued to increase and reached 2.63 mg/dL. Due to concern of worsening renal disease, avacopan therapy was prescribed, and within 2 days of avacopan initiation, SCr decreased and progressively normalized.

Following 10 months of avacopan therapy, the patient has been deemed to be in sustained disease remission. She has been tapered off methylprednisolone completely (Day 284). Steady renal improvement following avacopan-based regimen was observed. Renal function prior to initiating avacopan therapy was decreased, with the lowest recorded eGFR of 12 mL/min/1.73 m^2^. Following avacopan treatment, eGFR increased to 33 mL/min/1.73 m^2^ (SCr = 1.70 mg/dL) at approximately 1 month and to 55 mL/min/1.73 m^2^ (SCr = 1.15 mg/dL) at 10 months (Fig. 2B). Additionally, urinary protein:creatinine ratio, as an independent predictor of the longitudinal changes in eGFR [11], had precipitously dropped from the time of disease onset with an initial value of 5 g/g (moderate-grade proteinuria) to 1.7 g/g at 1 and 6 months, and to 0.5 g/g at 10 months (essentially low-grade proteinuria) after the initiation of avacopan (Fig. 2B). Furthermore, diabetes mellitus and hypertension have resolved and there have been no additional hospitalizations since initiation of avacopan. No adverse events or serious adverse events related to avacopan were noted.

## Discussion

Early disease diagnosis and innovative therapeutic approaches are critical for patients with ANCA-associated vasculitis. This patient case illustrates some challenges healthcare providers continue to face with the current standard of care treatments using immunosuppressants. Patients with ANCA-associated vasculitis may not achieve clinical remission, are at risk of early relapse, and may experience glucocorticoid-related adverse events and encounter disease flares with attempts to taper glucocorticoids.

The alternative complement pathway has been demonstrated to play an important role in ANCA-associated vasculitis [12]. In particular, the complement anaphylatoxin C5a, as a granulocyte, monocyte, and macrophage chemoattractant [13], is one of the most potent pro-inflammatory peptides that interacts with C5aR and primes neutrophils to enhance ANCA-induced neutrophil activation [14]. In addition to priming neutrophils, C5a enhances neutrophil retention in the microvasculature and promotes antigen recognition by T cells through activation of dendritic cells [15]. Several preclinical studies have confirmed the role of complement and the C5a/C5aR axis in causation of kidney damage, including proteinuria, renal fibrosis, and glomerulonephritis [16–21]. As a C5aR antagonist, avacopan prevented the development of glomerulonephritis induced by anti-myeloperoxidase antibodies in a murine model of ANCA-associated vasculitis [22]. These findings led to clinical investigations of avacopan that resulted in its approval by the FDA as an adjunctive treatment for adult patients with severe, active ANCA-associated vasculitis in combination with standard of care.

This patient did not achieve remission with B-cell mediated depletion therapies despite high dose glucocorticoid therapy. Following the addition of avacopan in combination with methylprednisolone (gradual taper starting at 50 mg), the patient had improvement in symptoms and kidney function and was able to achieve remission (with discontinued glucorticoid use, no additional hospitalizations, and resolution of diabetes mellitus and hypertension). While only a single case, this response may imply a synergistic role for C5aR blockade and B-cell mediated depletion therapies and suggests that C5a-mediated neutrophil activation and chemotaxis remains important as a B-cell independent pathway in the pathogenesis of ANCA-associated vasculitis. In ADVOCATE, rituximab was administered once weekly for 4 doses (in accordance with the rituximab label at the time), and additional prospective, long-term trials are needed to delineate the role of combination cytotoxic and chemotactic inhibitory therapy [23].

Most importantly, consistent with patient-reported outcomes from the ADVOCATE clinical trial [4], this patient has reported improvements to her quality of life. Avacopan provided this patient with a novel treatment option that appears to have improved the course of her ANCA-associated vasculitis disease.

The results from this case study and other published case series [7–10] suggest the potential for avacopan to: (1) achieve and sustain remission, (2) reduce glucocorticoid use, hospitalizations, and emergency room visits, and (3) aid in renal improvement and avoid dialysis. As the optimal treatment for refractory ANCA-associated vasculitis has not yet been thoroughly defined, further studies are needed to determine the potential role of avacopan in these cases.

## Data Availability

Data sharing requests, which should be directed to the corresponding author, will be reviewed upon request.
